# Characterization of Cetacean Proline-Rich Antimicrobial Peptides Displaying Activity against ESKAPE Pathogens

**DOI:** 10.3390/ijms21197367

**Published:** 2020-10-06

**Authors:** Riccardo Sola, Mario Mardirossian, Bertrand Beckert, Laura Sanghez De Luna, Dennis Prickett, Alessandro Tossi, Daniel N. Wilson, Marco Scocchi

**Affiliations:** 1 Department of Life Sciences, University of Trieste, 34127 Trieste, Italy; riccardo.sola@phd.units.it (R.S.); laura.sanghezdeluna@studenti.units.it (L.S.D.L.); dennis.pricketti@yahoo.it (D.P.); atossi@units.it (A.T.); mscocchi.units.it (M.S.); 2 Department of Medical Sciences, University of Trieste, 34125 Trieste, Italy; mmardirossian@units.it; 3 Institute for Biochemistry and Molecular Biology, University of Hamburg, 20146 Hamburg, Germany; beckert@chemie.uni-hamburg.de (B.B.); Daniel.Wilson@chemie.uni-hamburg.de (D.N.W.)

**Keywords:** *Cetacea*, antimicrobial peptide, proline-rich, cathelicidin, protein synthesis, membrane permeabilization, ESKAPE

## Abstract

Proline-rich antimicrobial peptides (PrAMPs) may be a valuable weapon against multi-drug resistant pathogens, combining potent antimicrobial activity with low cytotoxicity. We have identified novel PrAMPs from five cetacean species (cePrAMPs), and characterized their potency, mechanism of action and *in vitro* cytotoxicity. Despite the homology between the N-terminal of cePrAMPs and the bovine PrAMP Bac7, some differences emerged in their sequence, activity spectrum and mode of action. CePrAMPs with the highest similarity with the Bac7(1-35) fragment inhibited bacterial protein synthesis without membrane permeabilization, while a second subgroup of cePrAMPs was more membrane-active but less efficient at inhibiting bacterial translation. Such differences may be ascribable to differences in presence and positioning of Trp residues and of a conserved motif seemingly required for translation inhibition. Unlike Bac7(1-35), which requires the peptide transporter SbmA for its uptake, the activity of cePrAMPs was mostly independent of SbmA, regardless of their mechanism of action. Two peptides displayed a promisingly broad spectrum of activity, with minimal inhibiting concentration MIC ≤ 4 µM against several bacteria of the ESKAPE group, including *Pseudomonas aeruginosa* and *Enterococcus faecium*. Our approach has led us to discover several new peptides; correlating their sequences and mechanism of action will provide useful insights for designing optimized future peptide-based antibiotics.

## 1. Introduction

The emergence of antibiotic drug resistance across microbial species is acknowledged as a major global threat. Multi-, extended and even pan-drug-resistant resistant pathogens are spreading in both hospital and community environments due to extensive and continued misuse of antibiotics [1]. In this scenario, the acronym ESKAPE summarizes some of the most worrisome multi-drug resistant pathogens, i.e., *Enterococcus faecium*, *Staphylococcus aureus*, *Klebsiella pneumoniae*, *Acinetobacter baumannii*, *Pseudomonas aeruginosa* and *Enterobacter* spp. Clinicians are facing these bacterial species more and more, as they are often responsible of severe nosocomial infections, unfortunately resisting most of the current antibiotic therapies [2,3]. The preclinical antibacterial pipeline needs to be continually replenished by implementing novel approaches to tackle this problem [4,5]; many of these approaches often involve identification and characterization of naturally produced antimicrobial compounds to be developed as potential antibiotics [6].

A large number of antimicrobial peptides (AMPs), also known as host defense peptides (HDPs), have been identified as components of the innate immune systems of animals and plants. Despite difficulties in transiting through the clinical pipeline, AMPs have been widely recognized to have a major potential as anti-infective drug candidates [7,8,9,10]. This stems from their wide spectrum of activity and from mechanisms of action that are different to those of clinically relevant antibiotics, thus, they are considered to be less prone to be affected by microbial resistance(s) [11,12,13]. The mode of action of most AMPs involves destabilization and/or rupture of the bacterial membrane. In other words, AMPs tend to be membrane-directed antibiotics, which unfortunately very often correlates with toxicity profiles unsuitable for drug development [11]. However, some classes of AMPs have been identified that act on intracellular targets [14,15], as in the case of the proline-rich antimicrobial peptides (PrAMPs). At their relatively low active concentrations, these PrAMPs are taken up by susceptible bacterial cells (by membrane transporters, primarily SbmA [16] but also Yjil/MdtM [17]) and then interfere with the bacterial translational machinery [18,19,20,21]. This correlates with a narrower spectrum of activity for PrAMPs compared to membrane-directed AMPs, as they tend to target a narrower set of Gram-negative bacteria. This is most likely due to the requirement for the presence of the specific membrane transport systems reported above, as well as to their intracellular mode of action [22,23]. However, this limitation is overcome by some PrAMPs at higher concentrations, where they start to perturb the bacterial membrane and so switch to a membranolytic mechanism of action thereby displaying a broader spectrum of action [23,24]. In any case, this dual, concentration-dependent mechanism of action results in a generally lower toxicity towards eukaryotic cells—an advantage over those AMPs that are primarily membranolytic [25].

To date, all the PrAMPs discovered in mammalian species belong to the cathelicidins family of HDPs [23]. This comprises a heterogeneous group of AMPs that are synthesized as larger pro-forms in which the N-terminal pro-region (the function of which is still unclear) is well conserved across vertebrate animals, and from which the active peptide is released upon proteolytic cleavage [26,27]. Thus, while different cathelicidins vary quite significantly in their C-terminal antimicrobial moiety, their coding sequences share a common organization, with the first three exons coding for the conserved pro-region and the fourth exon coding for the variable, C-terminal AMP [25].

While most mammals express only one cathelicidin, all species of artiodactyls analyzed to date express several, and it is among these that the PrAMPs are found [28]. Cathelicidins expressed in bovids, ovids and pigs in particular, have been isolated and extensively characterized, including the well-known proline-rich ‘bactenecins’ Bac5 and Bac7 [23]. *Artiodactyla* and *Cetacea* are monophyletic, belonging to the clade of *Cetartiodactyla* [29,30], which led us to successfully mine the genome of the bottlenose dolphin *Tursiops truncatus* for cathelicidins, and identify the novel PrAMPs Tur1A and Tur1B [21]. These showed good antimicrobial properties against a reference strain of *Escherichia coli*, and Tur1A was also shown to inhibit protein synthesis, binding in the exit tunnel of the ribosome [21], as had previously been reported for active fragments of the longer bovine Bac7 [31,32]. It is of interest to note that this mechanism has also been reported for the evolutionarily distinct insect PrAMPs, such as oncocin-112, pyrrhocoricin, metalnikowin 1 [33,34] and to some extent also apidaecin [35], representing a clear case of convergent evolution to common structural motifs and a shared function; a good indication of a robust antimicrobial mechanism.

In this work we have continued this genome mining approach by looking for orthologs of the bovine and dolphin PrAMPs in the genomes of several other cetacean species, identifying five new sequences coded by five different cetacean species ranging from dolphins to orca, to whales.

These all conform to the canonical organization of the known cathelicidin genes, with a diversified fourth exon that corresponds to the putative C-terminal AMP moiety. These peptides are of similar size, and their sequences are clearly those of PrAMPs. We have explored the antimicrobial activity of these new cetacean PrAMPs (cePrAMPs) and have initiated the characterization of their mechanism of action, which appears to fall into two distinct types, with one group having the intracellular antimicrobial activity typical of PrAMPs and the other a mode of action that seems more membrane-directed. Subtle differences in their sequence arrangements are consistent with diversification into two functionally different subgroups. We deem that these molecules deserve more effort to define their properties, as they represent a fascinating evolutionary combinatorial experiment on how subtle changes in sequence motifs affect function. This may also provide useful criteria for optimization of these molecules as valid candidates to obtain new anti-infective drugs.

## 2. Results

### 2.1. Identification of New Cetacean PrAMPs

Searching for orthologs of the artiodactyl proline-rich cathelicidins in cetacean species, we screened for the cathelicidin gene clusters in publicly available databases by performing pBLAST searches on ‘non-redundant protein sequences’ in the NCBI database, using the pro-peptide of Bac7(1-35) as a query. We found five putative proline-rich peptide-coding sequences, each one in a different cetacean species (see Table 1); *Orcinus orca*, *Delphinapterus leucas*, *Balaenoptera acutorostrata*, *Lipotes vexillifer* and *Neophocaena asiaeorientalis.* These five sequences code for the distinctive N-terminal cathelin domain [25] and a diversified C-terminal region corresponding to a putative PrAMP. A previous search of the genome of *Tursiops truncatus* had found an analogous cathelicidin sequence (Tur1A), while a search in the EST databases revealed a second paralogous cathelicidin, also a PrAMP but with several Trp residues, termed Tur1B [21].

All the C-terminal sequences of these cetacean cathelicidins (putative PrAMPs) showed a significant degree of conservation amongst themselves, with similar lengths and arrangement of proline residues, and this extended to the well characterized 1-35 fragment of the bovine Bac7, in line with the monophyletic relationship of cetaceans and artiodactyls. This suggests that all these peptides derive from a common ancestral sequence. With the exception of Neo1, all the cetacean sequences have a length of 32 residues; the former sequence is only 19 residues long, but this is due to the presence of a premature stop codon and the significant sequence identity continues in the 3′-UTR region before a canonically placed second stop (see Table 1).

The cetacean PrAMPs (hereafter referred to as cePrAMPs) were systematically named by using the first three letters of the genus of the animal in which they were found. The proline content ranges between 30% and 34%, and all sequences show a high cationicity, ranging from +6 to +12, mostly due to arginine residues, with one conserved, C-terminal lysine residue (absent only in the cePrAMP Tur1A). The truncated Neo1 sequence (+3) is the exception, but it would be +6 were it not for the premature stop. Some of the peptides show a higher average hydropathy compared to Bac7(1-35), as indicated by the GRAVY index (obtained from the average of the hydrophobicity values of the sequence amino acids) (see Table 1). Two of the putative cePrAMP sequences, Bal1 and Lip1, are nearly identical to each other, aside for three conservative amino acid substitutions, and for the first 17 N-terminal residues are almost identical to Bac7(1-35) (see Table 1).

### 2.2. Antibacterial Activity—Membrane Directed or Internal?

We have investigated the antimicrobial properties of the new cePrAMPs and the possible reliance of their activity on their uptake through the inner membrane transporter SbmA, as observed for the majority of characterized PrAMPs of both artiodactyl or insect origin [23]. The peptides displayed significant inhibitory activity against a panel of reference bacterial pathogens and a clinical isolate, with MICs that in most cases were in the low micromolar range (Table 2 and Table S1 respectively), confirming that they are AMPs with an appreciable potency. With the exception of the truncated Neo1, all the other cePrAMPs displayed MIC values ≤ 8 µM against *E. coli*, *A. baumannii*, *Stenotrophomonas maltophilia* and the yeast *Candida albicans*. Bal1, Lip1 and Tur1A, which had the highest sequence similarity with Bac7(1-35) as well as a comparably high charge, were the most highly effective at inhibiting the growth of *E. coli, K. pneumoniae, A. baumannii, S. maltophilia*, *C. albicans* and a clinical strain of *Entrobacter agglomerans*, with MIC values comprised between 0.5 µM and 2 µM, similar to the bovine peptide.

It is worth noting that six full-length cePrAMPs to some extent also inhibited the growth of the Gram-positive bacterium *Staphylococcus aureus* (MIC = 8–32 μM), towards which Bac7 and other characterized PrAMPs of terrestrial mammals display poor activity (MIC > 64 μM). Bal1 and Lip1 showed the highest and broadest activity, with MIC ≤ 2 µM against all the Gram-negative pathogen and *C. albicans.* Their spectrum of activity is broader than the bovine PrAMP, with a potent inhibiting action also against *P. aeruginosa* and *E. faecium* (MIC = 2–4 µM), as well as an appreciable action against *S. aureus* (MIC = 16 µM)*,* all of which are bacterial species not normally susceptible to PrAMPs (Benincasa 2004, Scocchi 2011).

Neo1 displayed MICs ≥ 16 µM against almost every pathogen tested, suggesting that sequence truncation significantly weakened its antimicrobial potential. This is more likely to be due to its low cationicity, rather than its shorter size, as shortened fragments of Bac7 retaining high cationicity, such as Bac7(1-16) (+8), display an activity that is only slightly reduced with respect to longer fragments [36].

The peptides Orc1, Del1, Tur1B and Neo1 were difficult to resuspend in 100% Mueller-Hinton broth, possibly due to their higher hydrophobicity. To solve this issue and determine how it affected antimicrobial activity, bacterial susceptibility tests were repeated with most strains using 20% Mueller-Hinton broth. With some exceptions, these four peptides did indeed increase their activity in this medium, as did the others, in such a manner that the relative differences remained substantially unchanged (Table S1). Therefore, sequence-activity considerations remain valid.

We then evaluated the MIC against the *E. coli* BW25113 strain, for which a deletion mutant for the *sbmA* gene is available. This allows to test the relevance of the uptake through this transporter to the mechanism of action. While the MIC value of Bac7(1-35) against *E. coli* BW25113Δ*sbmA* was eight-fold higher than that against the wild type strain, surprisingly none of the cetacean peptides displayed an increase in MIC higher than two-fold in the absence of this membrane transporter (Table 2). Orc1 even showed a lower MIC against the *ΔsbmA* strain than the wild type. These observations could have two explanations; (i) either the cePrAMPs preferentially use a different transporter than SbmA or (ii) they have a radically different mode-of-action, involving cell penetration without transporters, or not requiring penetration at all (either way implying some form of membrane-directed action).

### 2.3. Dependence of cePrAMP Conformation on the Environment

The independence of cePrAMPs on the SbmA transporter for internalization, and the higher hydrophobicity shown by some of them with respect to Bac7(1-35), suggested that they might exert their action or at least part of it, directly on the bacterial membrane. This could enable them either to make the membrane their principal target, or to autonomously cross the membrane, and then inhibit cytosolic targets like other PrAMPs.

**Figure 1:**
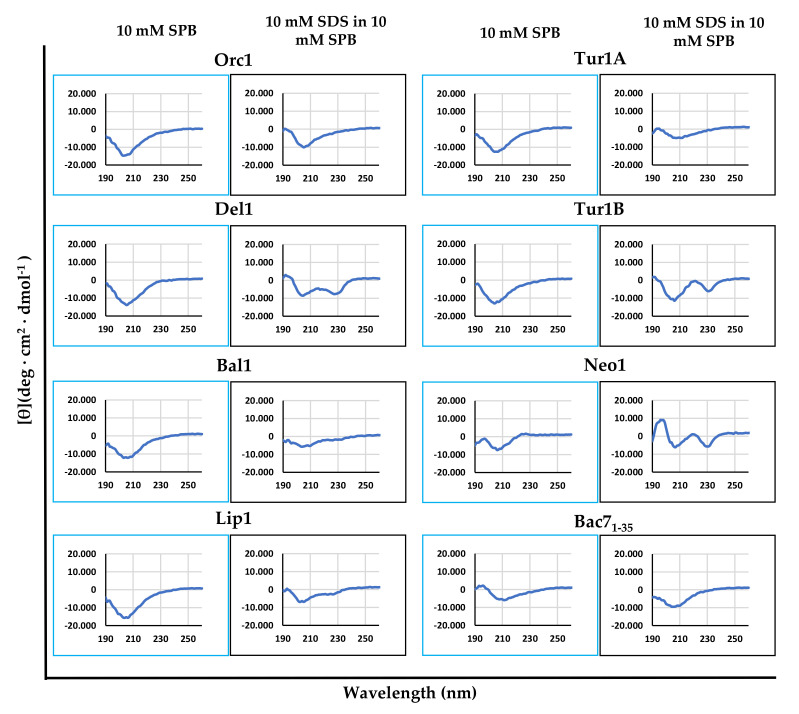
CD spectra of the peptides (20 µM) in 10 mM sodium phosphate buffer (SPB, first and third columns) and in 10 mM sodium dodecyl sulphate (SDS) in 10 mM SPB (second and fourth columns). The SDS concentration was above the critical concentration for forming micelles, to mimic the anisotropic bacterial membrane environment. Spectra derive from the accumulation of three scans.

Membrane-active AMPs generally undergo significant conformational changes when transiting from bulk solution into biological membranes, to optimize their amphipathicity and favor insertion into the membrane surface [37]. We investigated variations in the secondary structure of these peptides, by comparing their circular dichroism (CD) spectra in sodium-phosphate buffer (SPB) in the presence and absence of SDS micelles, to mimic the physiological aqueous environment and the anisotropic membrane surface (Figure 1). The cePrAMPs Del1, Tur1B and Neo1 showed a significant variation in their circular dichroism (CD) spectra in the presence of SDS micelles compared to only SPB, showing a second minimum around 230 nm, suggesting a propensity to interact and insert into biological membranes by altering their conformation to favor this process. The other peptides did not significantly alter their CD spectra, suggesting less interaction with SDS micelles, and by extension, also with membrane surfaces.

### 2.4. Membrane Permeabilization

Alterations in conformation upon membrane interaction is a hallmark of membranolytic AMPs. To determine whether the cePrAMPs have the capacity to permeabilize bacterial membranes, as suggested for some peptides based on the CD analysis, the β-galactosidase assay was performed on *E. coli* ML35p, in order to verify if permeabilization of the inner cytoplasmic membrane occurred in their presence. β-galactosidase can cleave the chromogenic probe dye *O*-nitrophenyl-β-d-galactopyranoside (ONPG), releasing the colored compound *O-*Nitrophenol; the ML35p strain of *E. coli* constitutively expresses this enzyme in its cytoplasm and is deficient of a sugar transporter, so that the impermeable ONPG cannot access the cytoplasm and reach the hydrolytic enzyme unless the cytoplasmic membrane is somehow breached [38]. The peptide antibiotic colistin, with a confirmed membranolytic mechanism of action, was used as a positive control for permeabilization.

**Figure 2:**
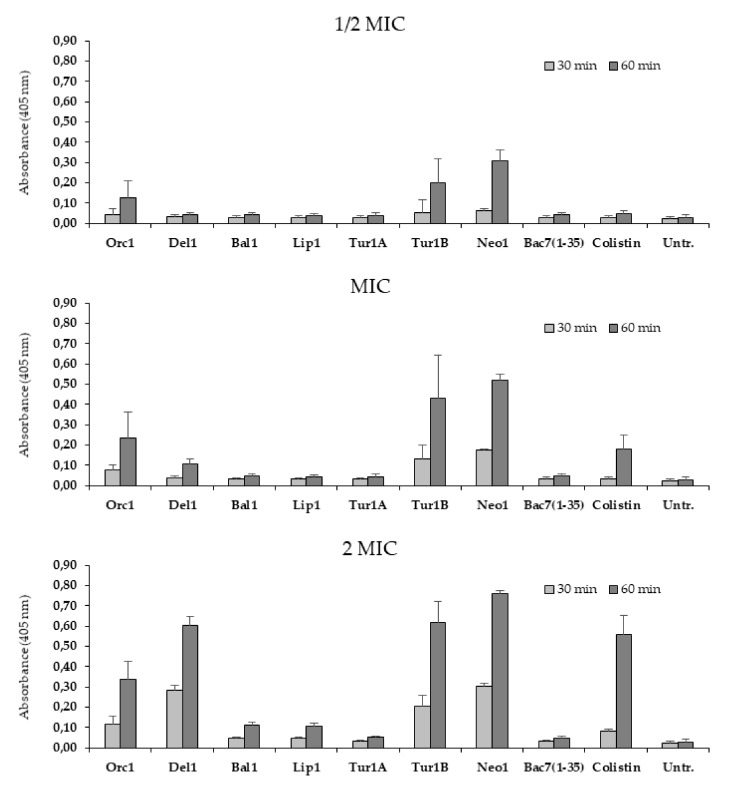
Cytoplasmic membrane permeabilization of *E. coli* ML35p by cePrAMPs. Peptide-mediated membrane permeabilization at ½ × MIC, 1 × MIC and 2 × MIC. Inner membrane permeabilization was monitored as an increase in absorbance at 405 nm by O-nitrophenol, a product of the hydrolysis of the impermeable, chromogenic substrate ONPG after 30′ and 60′ incubation. The membranolytic peptide antibiotic colistin has been used for comparison. The complete kinetics of permeabilization are shown in Figure S1.

The tested cePrAMPs were quite heterogenous in their membrane permeabilizing capacity and kinetics, which were tested at peptide concentrations corresponding to ½-, 1- and 2-fold of their MIC values (Figure 2), and this only partially correlated to their behavior in the CD analysis. Tur1B and Neo1, which showed marked changes in their CD spectra, increased the ONPG-mediated signal within 60 min of incubation, even at concentrations below their MIC values; however, so did Orc1, albeit to a lesser extent, even if its CD spectra did not change between polar and amphipathic environment. Del1, which also altered its CD spectrum markedly in the presence of SDS micelles, showed a slower but significant permeabilization at its MIC, but attained levels remarkably similar to Tur1B and Neo1 at 2 × MIC. All these peptides permeabilized the membrane to an extent similar to or higher than that of the membrane-active antibiotic colistin (Figure 2). On the contrary Bal1, Lip1 and Tur1A exhibited a very low permeabilizing effect, comparable to that of Bac7(1-35). Indeed, permeabilization induced by Bal1 and Lip1 at twice their MIC values was only marginally higher than the untreated control. These results highlighted the noticeable diversity between different cePrAMPs in interacting with, and affecting, the bacterial membrane.

### 2.5. Inhibition of Bacterial Protein Synthesis

**Figure 3:**
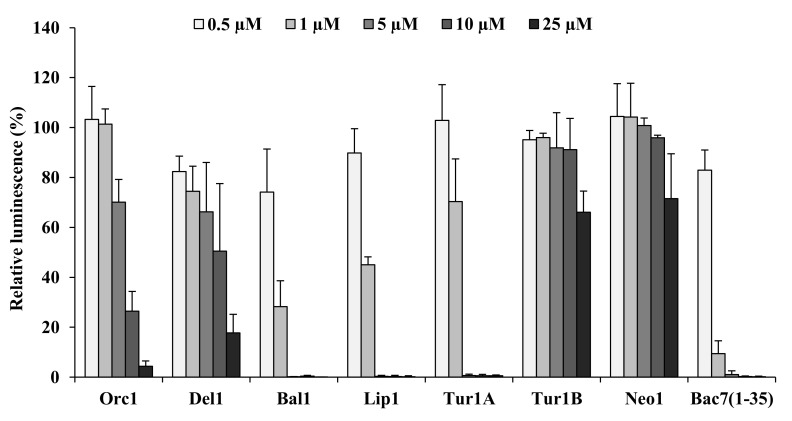
Luciferase activity after *in vitro* transcription/translation reactions in presence of cePrAMPs. *E. coli* extracts were incubated with the firefly luciferase reporter DNA in the presence of increasing concentrations of cePrAMPs. Results are presented as the percentage with respect to control samples treated with only RNase-free water. Average and standard deviation of at least three independent experiments (*n* ≥ 3).

Several PrAMPs derived from artiodactyl or insect peptides act by interfering with bacterial protein synthesis [19,34,39]. To evaluate if the cePrAMPs also targeted this process, *in vitro* transcription/translation assays were carried out in their presence, assessing the inhibition of the synthesis of a reporter luciferase (Figure 3). In line with what previously reported, Bac7(1-35) and Tur1A were both confirmed as potent inhibitors of protein synthesis [19,21,31], with active concentrations of 1–5 µM, while Tur1B barely affected the process and only at the highest tested concentration (25 µM). With respect to the newly identified cetacean PrAMPs, Bal1 and Lip1 also strongly reduced luciferase activity and therefore expression, to a level comparable to that of Bac7(1-35) and Tur1A.

Conversely, Orc1 and Del1 displayed only a moderate concentration-dependent inhibitory effect on the translation while Neo1 displayed almost no inhibitory activity on protein synthesis (Figure 3). These results suggested that while protein synthesis may be the prevalent target in the antibacterial mode of action of the peptides Bal1 and Lip1, this is not the case for Orc1, Del1, Tur1B and Neo1.

### 2.6. Evaluation of Potential Hemolytic and Cytotoxic Effects on Eukaryotic Cells

**Figure 4:**
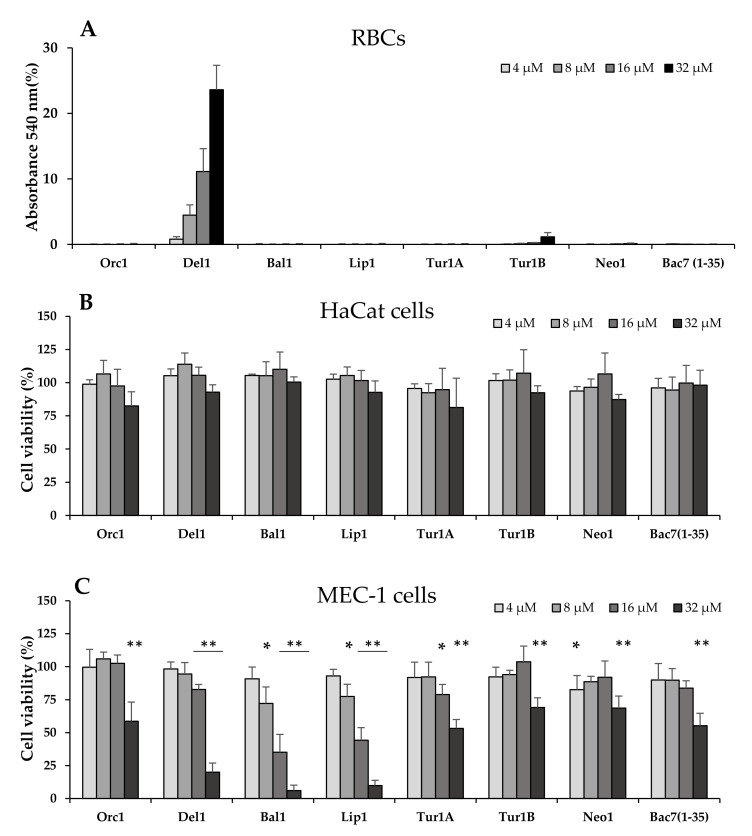
Evaluation of *in vitro* toxicity of CePrAMPs. (**A**) Hemolysis assay against human red blood cells (hRBCs), re-suspended in PBS at 4% (*v*/*v*) concentrations, measured as the absorbance of released hemoglobin (540 nm) after 1 h of exposure to the peptides. Results are reported as percentages with respect to hRBCs treated for 1 h with 1% Triton X-100 (considered as 100%), and are the average of three independent experiments (*n* = 3); (**B**,**C**) Evaluation via MTT assay of the viability of the indicated eukaryotic cell lines after exposure to the peptides. HaCat (**B**) or MEC-1 (**C**) cells were incubated with the different peptides at increasing concentrations for 21 h (**B**) or 20 h (**C**), before treating cells with MTT. Results are reported as the percentage of viable cells with respect to the untreated control, and are the average of three independent experiments (*n* = 3) (* = *p* < 0.05; ** = *p <* 0.01).

A particularly interesting aspect of PrAMPs, such as Bac7(1-35), is their very low cytotoxicity towards animal cells [40,41,42], which is highly relevant to the potential for development as an antibiotic for clinical use. To obtain a preliminary evaluation of the potential toxicity of cePrAMPs against host cells, they were assessed for their hemolytic effects on a suspension of 4% (*v*/*v*) human red blood cells (hRBCs) in PBS (Figure 4A), and with the exception of Del1, the peptides were all shown to be non-hemolytic. The assay was then repeated using a suspension of 0.4% hRBCs, to render the assay more sensitive, but this ended in essentially superimposable results (Figure S2). Apart from Del1, all the other cetacean peptides did not exhibit any hemolytic effect even at 32 µM.

Subsequently, the cePrAMPs were tested using the MTT assay for cytotoxicity towards the immortalized keratinocyte HaCaT cells and the tumoral lymphoid precursor MEC-1. In this case, none of the cePrAMPs displayed significant reduction of viability towards HaCat cells up to 32 µM peptide concentration (Figure 4B), indicating that cePrAMPs are non-toxic for these cells, with the exception of Orc1 at the highest tested concentration (32 µM, which however did not impair cell viability by more than 20% after 20 h incubation). The MEC-1 cells were instead generally more susceptible to the action of cePrAMPs (Figure 4B). In any case, cellular susceptibility became evident mostly at higher concentrations (8–16 µM or above), with Bal1 and Lip1 showing the most marked effects, reducing cell vitality by 20% and 50% at 8 and 16 µM, respectively (which corresponds to four–eight-fold of their MIC against most bacteria tested).

## 3. Discussion

Cathelicidins are one on the most widespread families of AMPs in vertebrate animals, and are characterized by a well-conserved N-terminal pro-region and a structurally varied C-terminal antimicrobial domain from which AMPs are proteolytically released. Among these, PrAMPs represent a distinctive subgroup defined by high contents of proline and arginine amino acid residues organized in many short repeats such as PRP or PPR. Most studies on mammalian PrAMPs have been performed on closely-related artiodactyl species including cow, sheep and other ruminants, in which the diversification of the C-terminal domains is relatively small, in line with short phylogenetic distances, so that peptides display similar activities and mechanisms of action (Scocchi 2011).

In this study we have characterized five new PrAMPs/cathelicidins from five different cetacean species, adding to another two recently identified sequences (Mardirossian et al., 2018). Despite a high sequence similarity, which clearly indicates that these are homologues to Bac7, they displayed significant differences in their mode of action and activity spectrum. For example, some of them possess a potent activity against bacterial species normally non-susceptible to most of the known native mammalian PrAMPs, including an appreciable activity against Gram-positive bacteria; thus, they have a wider spectrum of activity. Moreover, some of them seem to have switched to membrane permeabilization as the main mode of action. The comparative analysis of cePrAMPs sequences suggests a correlation between these features and particular sequence characteristics, which in turn points to the existence of two sub-groups of peptides; one putatively targeting protein synthesis but on a wider range of bacteria, and another affecting cell membranes with a distinct spectrum of activity.

Cetacean peptides with the highest sequence similarity with bovine Bac7(1-35), i.e., Bal1, Lip1 and Tur1A, displayed a potent antimicrobial effect but a markedly broader spectrum of activity than the bovine peptide. Bal and Lip showed a particularly broad spectrum that covered the whole group of ESKAPE pathogens, including a surprising MIC of ≤ 4 µM against the normally less susceptible *P. aeruginosa* and *E. faecium*, as well as an appreciable MIC of 16 µM against the normally PrAMP-resistant, Gram-positive pathogen *S. aureus*. This is despite the fact that their sequence and charge quite closely resemble that of Bac7(1-35). This suggests that it is possible to tweak the sequence of PrAMPs, especially at the C-terminal, to optimize their activity.

A clear distinction between the structure-function characteristics of the cetacean peptides was obtained by CD analyses. Peptides with relatively low charge combined with a higher hydrophobicity (gravy indexes) such as Del1, Tur1B and Neo, showed the highest conformational rearrangement in amphipathic environment. This corresponds to a more membranolytic mechanism of action, and could derive from the higher presence of Trp residues in the sequence. Nevertheless, the peptide Orc1, which does not markedly alter its conformation in presence of SDS micelles, represents a curious exception, and seems to exhibit two distinct mechanisms, at the membrane and cytosolic. In fact, while circular dichroism data fit nicely with the results obtained by bacterial membrane permeabilization assays for Del1, Tur1B and Neo, all displaying strongly modified spectra in the presence of SDS, Orc1 permeabilizes appreciably the membrane but without spectral alteration. This suggests that during their evolution, the sequence and mode of action of Orc1, Del1, Tur1B and Neo were altered to different extents from the mainly non-lytic effect of canonical PrAMPs to a more membrane-directed effect. Orc1 does not excel in either its membrane permeabilizing activity, nor its protein synthesis inhibiting activity, but both are quite measurable, thus, this dual activity may have been selected by evolution as a working compromise.

The transcription/translation inhibition data confirm this hypothesis, as Bal1, Lip1 and Tur1A, all non-membrane-permeabilizing, are strong inhibitors of protein synthesis, while membrane-permeabilizing cePrAMPs tend to be poorer inhibitors of protein synthesis in *E. coli*. However, unlike Tur1B, which seems to have switched all its translational inhibiting activity into a membranolytic one, Orc1 and Del1 seem to keep both mechanisms to some extent, with a stronger emphasis on membrane lysis. One possible explanation for this loss of inhibitory effect on translation by Orc1, Del1 and Tur1B may be sequence modifications affecting the “+XX(R/Y)LPRPRX” consensus motif, recently proposed by Mardirossian et al. [43] as crucial for PrAMPs inhibitory activity on translation, in accordance with previous studies on two insect-derived PrAMPs (oncocin [44] and pyrrhocoricin [45]).

The evolutionary trajectories that have led to the selection of two functionally distinct sub-groups of cePrAMPs in different cetacean species warrant a closer investigation in order to determine when this event occurred with respect to the differentiation of cetacean species from land-animals, and if possible, why. It may need to take into account the presence or absence of several other types of sequentially and functionally diverse cathelicidins in these species (Tossi et al., unpublished).

One curious and encouraging aspect is that the cePrAMPs with the highest permeabilizing activity were not necessarily the most toxic towards eukaryotic cells. In fact, only Del1 showed a relatively high hemolytic effect, while some peptides among the less permeabilizing PrAMPs, such as Bal1 and Lip1, exhibited some toxicity towards MEC-1 cells. The fact that the toxicity of cePrAMPs was rather dependent on the cell line suggests that PrAMP sequences offer an excellent opportunity for optimization. In fact, two of the cePrAMPs, Tur1B and Neo1, present rather interesting quandaries. They are among the more permeabilizing peptides when compared to the lytic AMP colistin, even at sub-MIC concentrations, and yet were among the least toxic. This also correlates with a relatively poor *in vitro* antimicrobial activity, thus, they require further investigation as to their actual functional roles in the host animal. In this respect, they are both to some extent anomalous, as Tur1B is the only second PrAMP found to date in a cetacean species, and Neo1 has been subject to a mutation event that introduced a premature stop codon.

It is also quite interesting that the antimicrobial activity of all cePrAMPs, even when acting internally, was found to be less dependent on the SbmA transporter than Bac7(1-35). This had already been observed previously for Tur1A (Mardirossian et al., 2018). Their mechanism of action is, therefore, somewhat different to that of the bovine peptide fragment, despite the relatively high sequence similarity (63% overall), especially considering the functionally important N-terminus (95% sequence identity in the first 17 residues of Bal1 and Lip1, see Table 1). This feature is highly relevant, as the loss of SbmA could be considered a potential resistance mechanism for bacteria susceptible to Bac7(1-35), but we show here that the loss of this protein is evidently not a successful resistance strategy for bacteria against even closely related PrAMPs.

In conclusion, we have found a number of cetacean peptides with interesting antibiotic activity towards several pathogens, including the ESKAPE group, and their characterization has distinguished two distinct subgroups of sequences with quite different properties and mechanism of action, despite clearly correlated sequences. They represent a fascinating natural combinatorial experiment, where two quite distinct bacterial inactivation mechanisms, internalization and inhibition of protein synthesis or membrane lysis, have been used as the selective processes. Further studies of their properties might reveal interesting relations between sequence motifs, structures, toxicity and stability. These data would enable strategies of sequence optimization and combinatorial use that could prove useful for the development of anti-infective agents to treat different types of infections.

## 4. Materials and Methods

### 4.1. Peptides

Peptide sequences were identified by first performing pBLAST in the NCBI Non-Redundant Protein Sequence database with the sequence of the Bac7 cathelicidin, and subsequently accessing the NCBI Nucleotide sequence database, or if the first approach was not successful, blasting the NCBI Nucleotide database directly with the Bac7 nucleotide sequence. The complete pro-form nucleotide sequence was then downloaded and analyzed using the Artemis software [46] which allowed to verify the canonical cathelicidin intron/exon arrangement and extract the sequence of the 4th exon (the PrAMP).

All the peptides corresponding to the identified PrAMPs were purchased from NovoPro Bioscience Inc. (Shanghai, China), where they were synthesized via solid-phase FMOC chemistry and purified by reversed-phase HPLC to a purity of ≥95%, after which the correct primary structure was verified by mass spectrometry. The lyophilized peptides had trifluoroacetate (TFA-) as a counter-ion, which was substituted by the more biocompatible chloride (Cl-) counter-ion by resuspending in 10 mM HCl and lyophilizing three times. After the third lyophilization, the peptides were then resuspended in sterile milliQ water and quantified using a NanoDrop 2000 spectrophotometer. Briefly, the absorbance of peptide solution at 214 nm was measured, the molar extinction coefficient of the peptides was calculated using the guidelines from a work of of Kuipers and Gruppen [47], and the Lambert-Beer law was utilized to calculate the peptide concentrations. All the peptides were stored frozen, at −20 °C.

### 4.2. Microbial Strains

The following microbial reference strains were utilized: *Escherichia coli* BW25113, *E. coli* ATCC 25922, *E. coli* ML35p, *Enterococcus faecium* ATCC 19434, *Staphylococcus aureus* ATCC 25923, *Klebsiella pneumoniae* ATCC 700603, *Acinetobacter baumannii* ATCC 19606, *Pseudomonas aeruginosa* ATCC 27853, *Stenotrophomonas maltophilia* ATCC 13637, *Candida albicans* ATCC 90029. The *ΔsbmA* strain of *E. coli* BW25113 belongs to the KEIO collection [48] and the clinical isolate of *Enterobacter agglomerans* was kindly provided by Prof. C. Lagatolla from the Microbiology Laboratory of the University of Trieste. All the growth media were purchased from Difco (part of Thermo Fisher Scientific, MA, USA).

Bacteria were grown in Mueller-Hinton broth (MHB), except *E. faecium*, which was grown overnight in brain-heart infusion (BHI); *E. coli* BW25113 *ΔsbmA* was grown in the presence of 50 μg/mL kanamycin. *C. albicans* was grown in Sabouraud broth.

All the strains were grown overnight at 37 °C (except *E. agglomerans, S. maltophilia* and *C. albicans*, which were grown at 30 °C), with shaking at 140 rpm. The overnight bacterial cultures were then diluted approximately 1:40 in fresh growth medium and incubated at 37 °C or 30 °C with shaking (140 rpm) until an OD600 of between 0.3 and 0.5 was reached.

### 4.3. Circular Dichroism (CD) Spectra

Peptides were dissolved to a final concentration of 20 µM in 10 mM Sodium Phosphate Buffer (SPB pH 7.3), or in a solution of 10 mM Sodium Dodecyl-Sulphate (SDS) in 10 mM SPB, (pH 7.3). Peptides were dispersed into the solutions in quartz cells (0.1 cm path length) and the dichroism spectra were rapidly measured on a J-715 spectropolarimeter (Jasco, Easton, MD, USA), scanning from 190–260 nm at a speed of 50 nm/min, data pitch 0.5 nm, band width 1.0 nm. All displayed CD spectra are the accumulation of three scans, and were then smoothed using the tool provided by the Jasco software package.

### 4.4. Minimum Inhibitory Concentration (MIC) Determination

The minimum inhibitory concentration was determined by the broth microdilution method according to the NCCLS protocol. Briefly, an overnight bacterial or fungal culture was diluted in fresh MH or Sabouraud broth, respectively (300 µL O/N culture in 10 mL MHB/Sabouraud) and the culture was let grow at 37 °C or 30 °C to reach OD600 between 0.3 and 0.5. Serial two-fold dilutions of peptides were then performed in culture medium (Sabouraud, MHB or 20% MHB in PBS), to a final volume of 50 µL in the wells of a round bottom microtiter plate (Sarstedt, Milan, Italy), and then the bacterial suspension with a load of 5 × 10^5^ CFU/mL was added to the plate, halving the final concentration of bacteria and peptides. The culture was grown at 37 °C or 30 °C and the MIC was determined after 18h by visual inspection as the first clear well. The assessment was performed taking into consideration at least three independent experiments performed in duplicate.

Before the permeabilization assays, the MIC of PrAMPs and colistin against *E. coli* ML35p was determined with the same method but using a bacterial suspension with a load of ~2 × 10^7^ CFU/mL (=1 × 10^6^ CFU/well), so as to match the bacterial density of the permeabilization assays.

### 4.5. Assessment of Bacterial Membrane Permeabilization

Peptides were diluted in PBS into the wells of a 96-well transparent flat bottom microtiter plate (Sarstedt, Milan, Italy) to reach concentrations corresponding to ½, 1, and 2× their MIC values, as determined in the broth microdilution assay using 20% MH in PBS. Subsequently, a mid-log phase culture of *E. coli* ML35 was diluted in PBS, and *O*-nitrophenyl-β-d-galactopyranoside (ONPG) was added so as to reach a bacterial density of 2 × 10^7^ CFU/mL and ONPG concentration of 3 mM. This suspension was then dispensed to the wells of the microtiter plate (10^6^ CFU/well). Immediately after adding bacteria the plate was placed at 37 °C into a pre-heated Nanoquant infinite M200pro plate reader (Tecan, Männedorf, Switzerland), and the absorbance at 405 nm of the ONPG metabolite (O-nitrophenol) was measured every 10 min, after an initial plate shaking (10 s of 2 mm diameter—orbital shaking, 57 rpm).

### 4.6. In Vitro Transcription/Translation Assay

Reactions were set up using lysates of the RTS 100 *E. coli* HY kit from Biotechrabbit (Berlin, Germany) according to the user manual. Briefly, 1 μL of peptide solution was added to each reaction to reach the desired final concentration in a final volume of 6 μL. Samples were incubated at 30 °C for 1 h with shaking (600 rpm), then 2 μL of each reaction were extracted and stopped using 8 μL kanamycin solution (50 µg/mL) before being combined with 40 μL of Luciferase Assay Reagent (Promega, Madison, WI, USA) in a 96-well white flat bottom microtiter plate (Sarstedt, Milan, Italy). The presence of luciferase reporter protein was quantified by measuring its luminescence using a Infinite M200 plate reader (Tecan, Männedorf, Switzerland). The relative luminescence values for all the assays were calculated as a percentage of the positive control (reactions with nuclease-free water instead of the peptides) using three independent replicates. In the negative control, nuclease-free water was added instead of peptides and DNA template.

### 4.7. Determination of Cytotoxicity

#### 4.7.1. Hemolysis Assay

The lytic potential towards eukaryotic cell membranes was assessed by measuring the release of hemoglobin from human red blood cells (hRBCs) after exposure to the peptides. hRBCs were collected from single donor- human whole blood (Cambridge Bioscience, UK), being isolated by centrifugation, washed three times with PBS, and resuspended to either 8% or 0.8% (*v*/*v*) in PBS. Serial two-fold dilutions of the antimicrobial peptides in PBS were made in a 96-well flat bottom microtiter plate (Sarstedt, Milan, Italy), to a volume of 100 µL. Subsequently, 100 µL of either 8% or 0.8% hRBCs suspension were added to every well (final concentrations = 4% and 0.4%) and the plate was incubated for 1 h at 37 °C, then centrifuged at 1000× *g* for 10 min. Then, aliquots of 100 μL of supernatant were transferred to a new 96-well transparent flat bottom microtiter plate, and the release of hemoglobin was measured by reading the absorbance at 540 nm with a Nanoquant infinite M200pro plate reader (Tecan). The release of hemoglobin by hRBCs treated with PBS or 1% *v*/*v* Triton X-100, was taken as corresponding to 0% and 100% hemolysis, respectively.

#### 4.7.2. MTT Cytotoxicity Assay

The effect of peptide exposure on cell vitality was measured using the MTT assay on a human B lymphocytes precursors cell line (MEC-1) and on an immortalized human keratinocytes cell line HaCat.

In the MTT assay against MEC-1 cells, serial two-fold dilutions of the peptides were prepared in final 50 µL of complete RPMI1640 medium in a 96-wells transparent flat bottom microtiter plate, specially treated for tissue-culture (Euroclone, Milan, Italy). Cells were counted in a Burker-Türk Chamber, and then 50 µL of a diluted cell suspension (2 × 10^6^ cells/mL) were added to each well of the plate, halving the final cell density and peptide concentrations. The plate was incubated for 20 h at 37 °C in 5% CO_2_. Subsequently, 25 µL of MTT, dissolved in Phosphate Buffered Saline (PBS), were added to each well to the final concentration of 1 mg/mL MTT, and the plate was incubated 4 h in the dark at 37 °C in 5% CO_2_. At the end of the incubation, 100 µL of 10% Igepal (*w*/*v*) in 10 mM HCl were added to each well, and the plate was incubated overnight, in the dark at 37 °C, 5% CO_2_.

In the MTT assay against HaCat cells (growing in adhesion), cells were first counted in a Burker-Türk Chamber and diluted to 2.2 × 10^5^ cells/mL. 90 µL of such cell suspension were seeded in a 96-wells transparent flat bottom microtiter plate specially treated for tissue-culture (Euroclone, Milan, Italy), to a final density of 2 × 10^4^ cells/well. The plate was incubated 24 h at 37 °C with 5% CO_2_, before any treatment. After this incubation, two-fold serial dilutions of the peptides were prepared in PBS and added to the wells, to reach the desired concentrations in a final total volume of 100 µL/well. The plate was incubated for 21 h at 37 °C, 5% CO_2_, after which 25 µL of MTT solution in PBS were added to a final concentration of 1 mg/mL, and the plate was incubated for 3 h in the dark at 37 °C, 5% CO_2_. The plate was then centrifuged at 2000× *g* for 10 min, and the supernatant from each well was then substituted with 125 µL of PBS + 100 µL of 10% Igepal (*w*/*v*) in 10 mM HCl. The plate was incubated overnight in the dark at 37 °C, 5% CO_2_.

For both the cell lines, the MTT absorbance at 570 nm was measured in a Nanoquant Infinite-M200Pro plate reader (Tecan, Männedorf, Switzerland). As a statistical analysis, Student’s T-test was performed.

## Figures and Tables

**Table 1:** Sequence and physico-chemical properties of cetacean cePrAMPs, identified by genome mining, compared to the active N-terminal fragment of the bovine PrAMP Bac7(1-35).

Species	Peptide	Sequence ^a^	q ^b^	H(GI) ^c^
*Orcinus orca*	Orc1	RRIPFWPPNLPGPRRPPWFLPDFRIPRIPRKR	+8	−1.1
*Delphinapterus leucas*	Del1	RRIPFWPIPLRWQWPPPWFPPSFPIPRISRKR	+7	−0.8
*Neophocaena asiaeorientalis*	Neo1 ^d^	RRIRFPFPPFPWQWPPAGF*ptfhipriprkq *	+3	−0.6
*Tursiops truncatus*	Tur1A	RRIRFRPPYLPRPGRRPRFPPPFPIPRIPRIP	+10	−1.1
	Tur1B	RRIPFWPPNWPGPWLPPWSPPDFRIPRILRKR	+6	−1
*Balaenoptera acutorostrata*	Bal1 ^e^	RRIRFRPPRLPRPRPRPWIPPRFPFPRIPGKR	+12	−1.5
*Lipotes vexillifer*	Lip1 ^e^	RRIRIRPPRLPRPRPRPWFPPRFPIPRIPGKR	+12	−1.4
*Bos taurus*	Bac7(1-35)	RRIRPRPPRLPRPRPRPLPFPRPGPRPIPRPLPFP	+11	−1.4

^a^ Residues that are conserved in >50% of sequences are shaded grey; ^b^ peptide charge; ^c^ average *hydrophobicity* (GRAVY index score); ^d^ part of the 3′-UTR region of Neo1, just after the premature stop codon, is shown in lowercase (* = stop codon). ^e^ A highly identical N-terminal stretch between Bal1 and Lip1 cePrAMPs and Bac7 is underlined.

**Table 2:** MIC values for cePrAMPs, compared to bovine Bac7(1-35), against a panel of reference pathogenic bacterial and fungal strains.

Microorganism and Strain	MIC * (µM)							
	Orc1	Del1	Bal1	Lip1	Tur1A	Tur1B	Neo1	Bac7_1-35_
*E. coli* ATCC 25922	6	6	1	1	1	8	16	1
*E. faecium* ATCC 19434	16	4	4	4	64	16	>64	64
*S. aureus* ATCC 25923	*32*	8	16	16	>64	32	16	>64
*K. pneumoniae* ATCC 700603	32	>64	1	1	2	>64	>64	2
*A. baumannii* ATCC 19606	2	4	1	1	1	4	16	2
*P. aeruginosa* ATCC 27853	32	16	2	2	16	32	64	16
*E. agglomerans—clinical isolate*	8	8	0.5	0.5	1	16	64	0.5
*S. maltophilia* ATCC 13637	2	4	1	0.5	1	8	24	1
*C. albicans* ATCC 90029	4	8	1.5	2	2	8	32	2
*E. coli* BW25113	8	8	1	1	0.75	16	24	0.75
*E. coli* BW25113—*Δ SbmA*	2	8	2	1.5	1.5	8	16	8

* Results are reported as the median of 3 or more independent experiments (*n* ≥ 3).

## Supplementary Materials

Supplementary materials can be found at https://www.mdpi.com/1422-0067/21/19/7367/s1.

## Abbreviations

AMPs	Antimicrobial peptides
ESKAPE	*Enterococcus faecium, Staphylococcus aureus, Klebsiella pneumoniae, Acinetobacter baumannii, Pseudomonas aeruginosa, Enterobacter* spp.
cePrAMPs	Cetacean proline-rich antimicrobial peptides
GRAVY	Grand average of hydropathy
ESTs	Expressed sequence tags
MIC	Minimum Inhibitory concentration
CD	Circular dichroism
SPB	Sodium phosphate buffer
SDS	Sodium dodecylsulphate
ONPG	O-Nitrophenyl-β-D-galactopyranoside
MTT	3-(4,5-dimethylthiazol-2-yl)-2,5-diphenyltetrazolium (bromide)
