# Supplementary material for: Characterization of Cetacean Proline-Rich Antimicrobial Peptides Displaying Activity against ESKAPE Pathogens
Source: Int J Mol Sci. 2020 Oct 6;21(19):7367. doi: 10.3390/ijms21197367 (PMC7582929; doi:10.3390/ijms21197367)
Supplement: Supplementary file 1 [file ijms-21-07367-s001.zip › Revised Supplementary figures/Fig. S1 (Revised) + caption.pdf]

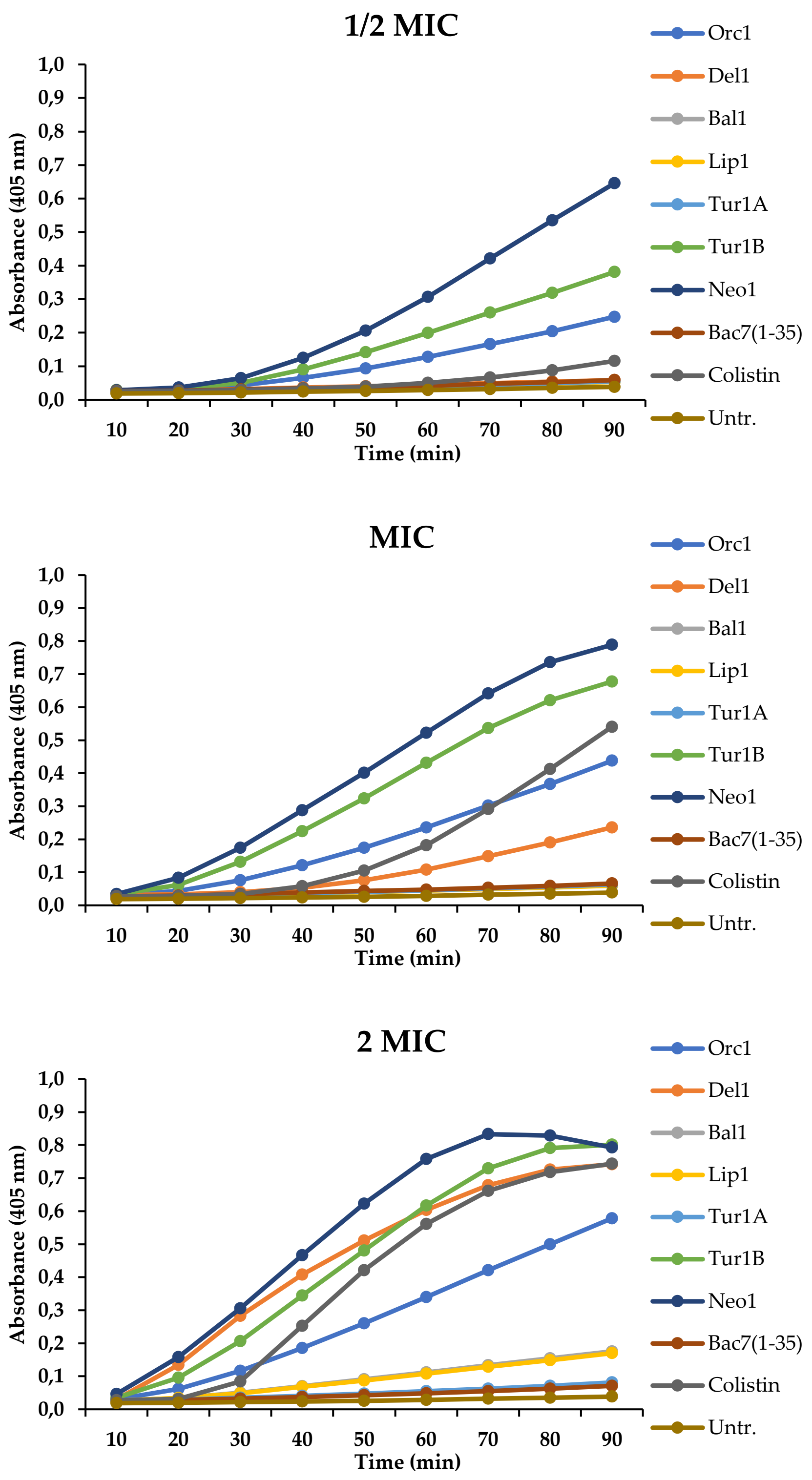

Figure S1. Kinetics of peptide-induced permeabilization of bacterial membrane. The absorbance at 405 nm of the ONPG hydrolysis product O-nitrophenol, was measured every 10 minutes for a total of 90 minutes. The membranolytic peptide antibiotic colistin has been used for comparison.
