# Supplementary material for: Characterization of Cetacean Proline-Rich Antimicrobial Peptides Displaying Activity against ESKAPE Pathogens
Source: Int J Mol Sci. 2020 Oct 6;21(19):7367. doi: 10.3390/ijms21197367 (PMC7582929; doi:10.3390/ijms21197367)
Supplement: Supplementary file 1 [file ijms-21-07367-s001.zip › Revised Supplementary figures/Fig. S2 (Revised) + caption.pdf]

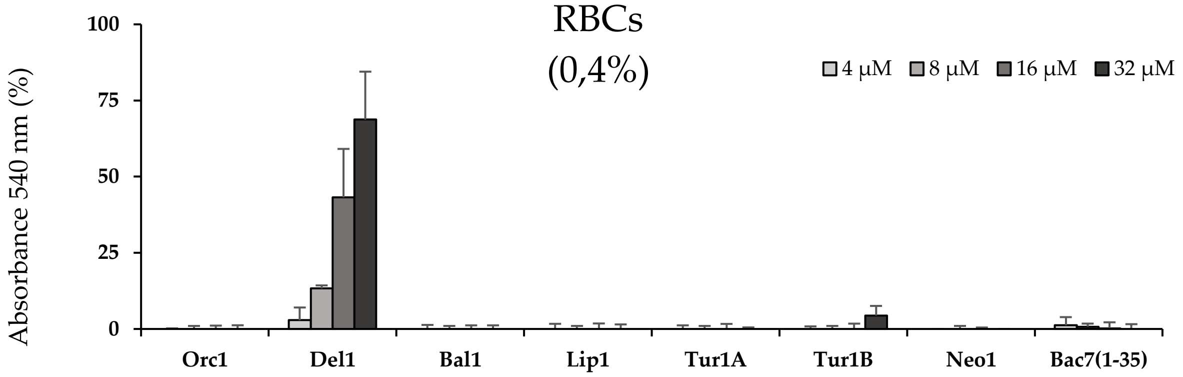

Figure S2. Hemolysis assay against human red blood cells, re-suspended in PBS at 0,4% (v/v) concentration. Hemolysis was measured as the absorbance of released haemoglobin (540 nm) after 1h exposure to the peptides. Results are reported as percentages with respect to hRBCs treated for 1h with 1% Triton X-100 (considered as 100% haemolysis), and are the average of 2 independent experiments (n=2).
