# Supplementary material for: Characterization of Cetacean Proline-Rich Antimicrobial Peptides Displaying Activity against ESKAPE Pathogens
Source: Int J Mol Sci. 2020 Oct 6;21(19):7367. doi: 10.3390/ijms21197367 (PMC7582929; doi:10.3390/ijms21197367)
Supplement: Supplementary file 1 [file ijms-21-07367-s001.zip › Revised Supplementary figures/Tab. S1 + caption.pdf]

Table S1. MIC of cePrAMPs in 20% Mueller-Hinton broth in PBS.

Microorganism and strain	MIC* (μM)							
	Orc1	Del1	Bal1	Lip1	Tur1A	Tur1B	Neo1	Bac7(1-35)
E.coli **BW25113**	4	1	0.5	0.5	0.5	4	16	0.5
E.coli **BW25113 ΔSbmA**	2	1	0.5	0.5	1	4	8	2
E.coli **ATCC 25922**	4	2	0.5	0.5	0.5	4	32	0.5
S.aureus **ATCC 25923**	64	2	4	4	>64	16	64	>64
K. Pneumoniae **ATCC 700603**	64	8	0.5	0.5	1	>64	>64	1
A. Baumannii **ATCC 19606**	1	1	0.5	0.5	0.5	1	8	0.5
P. aeruginosa **ATCC 27853**	16	8	1	1	4	16	64	2

* Results are reported as the median of 3 or more independent experiments (n ≥ 3).
