# Supplementary figures and images for: Characterization of Cetacean Proline-Rich Antimicrobial Peptides Displaying Activity against ESKAPE Pathogens
Source: Int J Mol Sci. 2020 Oct 6;21(19):7367. doi: 10.3390/ijms21197367 (PMC7582929; doi:10.3390/ijms21197367)

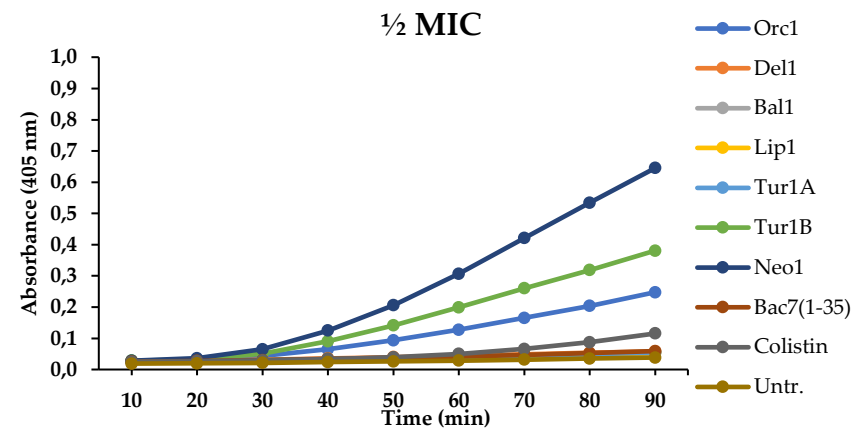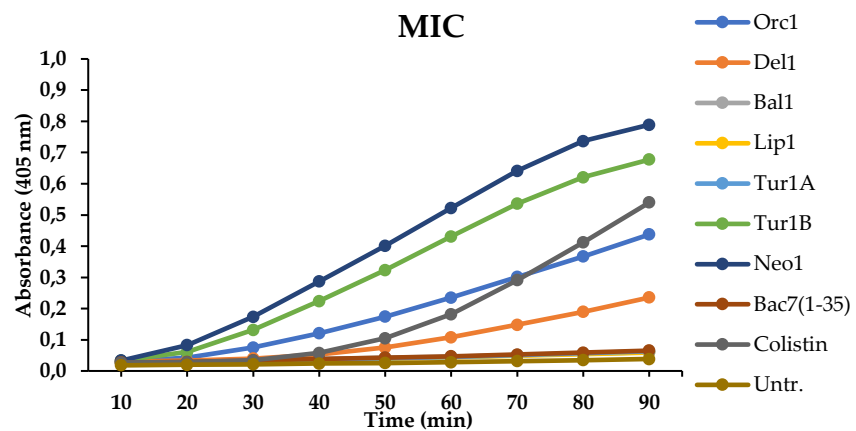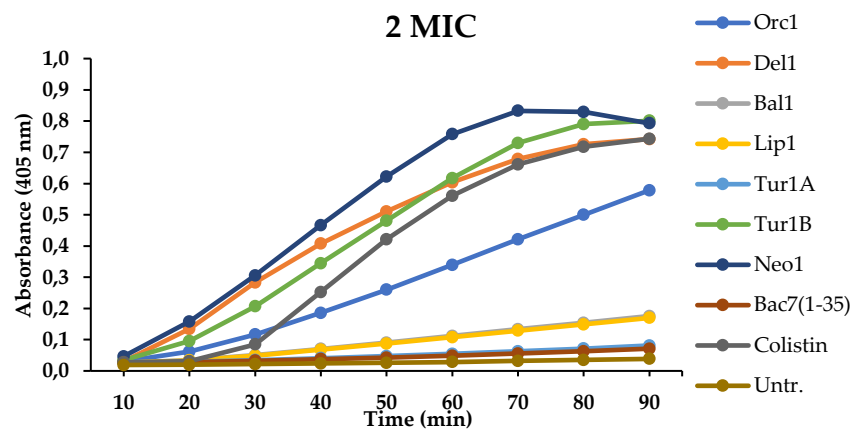

Supplement: Supplementary file 1 [file ijms-21-07367-s001.zip › Revised Supplementary figures/Fig. S1 (Revised).pdf]

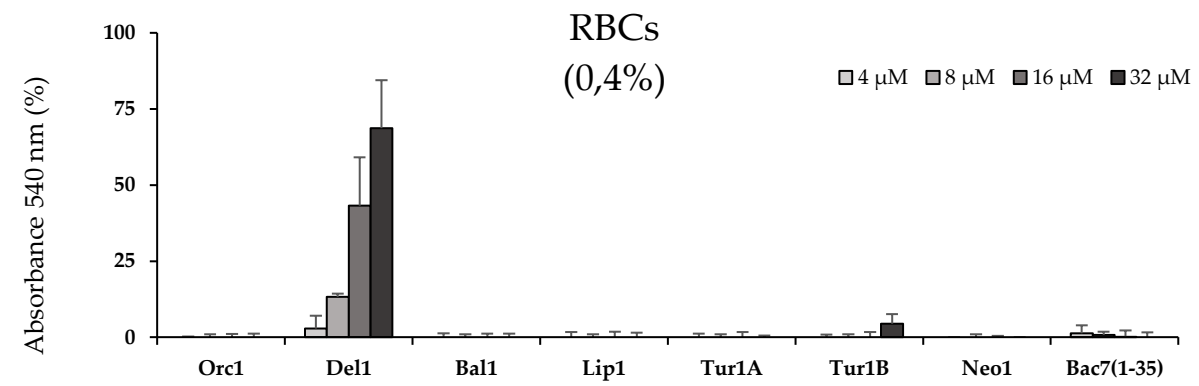

Supplement: Supplementary file 1 [file ijms-21-07367-s001.zip › Revised Supplementary figures/Fig. S2 (Revised).pdf]
